# Fixed-Precision Sequential Sampling Plans for Estimating Alfalfa Caterpillar, *Colias lesbia*, Egg Density in Alfalfa, *Medicago sativa*, Fields in Córdoba, Argentina

**DOI:** 10.1673/031.013.4101

**Published:** 2013-05-13

**Authors:** Gerardo V. Serra, Norma C. La Porta, Susana Avalos, Vilma Mazzuferi

**Affiliations:** Catedra de Zoología Agrícola, Facultad de Ciencias Agropecuarias, Universidad Nacional de Córdoba, Av. Valparaiso s/n, 5000 Córdoba, Argentina

**Keywords:** enumerative sampling, Green's model, Kuno's model, spatial distribution, resampling validation

## Abstract

The alfalfa caterpillar, *Colias lesbia* (Fabricius) (Lepidoptera: Pieridae), is a major pest of alfalfa, *Medicago sativa* L. (Fabales: Fabaceae), crops in Argentina. Its management is based mainly on chemical control of larvae whenever the larvae exceed the action threshold. To develop and validate fixed-precision sequential sampling plans, an intensive sampling programme for *C. lesbia* eggs was carried out in two alfalfa plots located in the Province of Córdoba, Argentina, from 1999 to 2002. Using Resampling for Validation of Sampling Plans software, 12 additional independent data sets were used to validate the sequential sampling plan with precision levels of 0.10 and 0.25 (SE/mean), respectively. For a range of mean densities of 0.10 to 8.35 eggs/sample, an average sample size of only 27 and 26 sample units was required to achieve a desired precision level of 0.25 for the sampling plans of Green and Kuno, respectively. As the precision level was increased to 0.10, average sample size increased to 161 and 157 sample units for the sampling plans of Green and Kuno, respectively. We recommend using Green's sequential sampling plan because it is less sensitive to changes in egg density. These sampling plans are a valuable tool for researchers to study population dynamics and to evaluate integrated pest management strategies.

## Introduction

Alfalfa, *Medicago sativa* L. (Fabales: Fabaceae), is a key resource for agricultural production in temperate regions worldwide. The area cultivated with alfalfa in Argentina is about five million hectares, one million of which are in the province of Córdoba, where forage is essential for milk and meat production. Alfalfa is also important for the sustainability of farming through its action in the recovery of soil fertility and stability (Pordomingo 1995).

Larvae of several lepidopteran species, such as *Colias lesbia* (Fabricius) (Pieridae), *Spodoptera frugiperda* (Smith), and *Rachiplusia nu* (Guenée), develop in and consume the leaves of alfalfa, the most valuable part of the plant. The biology of one of the key pests, *C. lesbia,* also known as the alfalfa caterpillar, has been largely documented (Freiberg 1947; Molinari 1968; Aragón and Imwinkelried 1995). Each female moth lays about 200 to 300 isolated eggs, typically on the upper surface of leaves (Aragón and Imwinkelried 1995), with eight to nine generations per year in the central region of the Pampas (Aragón and Harcourt 1977a). Only two or three of these generations cause severe damage to alfalfa crops from December to March, producing losses that can equal one or two cuts of forage in such rich production areas as northern Buenos Aires, La Pampa, Entre Ríos, Córdoba, and Santa Fe (Harcourt et al. 1980; Imwinkelried et al. 1992).

Currently, management is based on cultural or chemical control methods when larvae density exceeds the economic threshold of 0.6 larvae/stem (Harcourt et al. 1980). Cultural control consists in a cutting or heavy grazing, which promote the conservation of natural enemies but can be applied only once in a season, and plant height at the time of the pest attack may be inappropriate for a cutting. Chemical control may use a biological insecticide formulated from *Bacillus thuringiensis* (Bt), or synthetic chemicals, such as endosulfan, organophosphates, and pyrethroids. Bt insecticide does not affect natural enemies, but broad spectrum synthetic chemicals can be used at low doses with reduced negative effects on natural enemies, because *C. lesbia* larvae are highly susceptible (Harcourt et al. 1980; Aragon and Imwinkelried 1995). The use of low doses is not always complied with, however. Accordingly, Imwinkelried and Aragon (1995) recommended the inundative release of egg parasitoids as a complementary practice that not only has low impact on the environment, but is also consistent with the aims of integrated pest management. However, in Argentina there is little research in this area.

Integrated pest management is based, among other aspects, on the development of efficient sampling schemes and the knowledge of economic thresholds (Gyenge et al. 1999). Reliable and cost-effective sampling methods are critical for pest management and an important contribution to research into population ecology and population dynamics. Validation and evaluation of these methods are central to their development and implementation in the field (Naranjo and Hutchison 1997). A basic requirement for sampling plan development is knowledge of the spatial distribution of the insect (Taylor 1984). However, dispersion patterns of *C. lesbia* eggs have been little studied in Argentina, despite the importance of this pest in alfalfa (Aragón and Harcourt 1977b). In a previous publication, the authors examined the distribution pattern of *C. lesbia* eggs in alfalfa fields (Serra et al. 2005), but no appropriate sampling plans for estimating their abundance in the field had been developed. The aim of this paper was to develop and validate fixed precision sequential sampling plans to estimate *C. lesbia* egg density in alfalfa fields.

## Methods and Materials

### Data Collection

The study was conducted in two alfalfa fields in the province of Córdoba, Argentina, 50 km apart. One of the fields (3 ha in area) was in the experimental farm of the Faculty of Agricultural Sciences of the Córdoba National University (FCA), latitude 31° 29′ S, longitude 64° 00′ W; the other field (15 ha) was at the INTA Manfredi Experimental Station (Manfredi), latitude 31° 49′ S, longitude 63° 46′ W. The average annual rainfall is 759 mm, and average annual temperature is 16.8° C. Both fields were planted with alfalfa variety INTA Monarca and were not fertilized. Weed control was with herbicides before planting, and cuts were made approximately every 35 to 50 days, depending on weather conditions, for a total of 7 to 8 annual cuts. No insecticides were applied during the study. The sampling area was 1 ha in each site. Samplings were conducted at both sites weekly in spring and summer and biweekly in autumn and winter from 1999 to 2002. Every time the alfalfa was cut, sampling was suspended for two weeks in that field. A total of 78 data sets were collected during the study, 42 at FCA and 36 at Manfredi. The sampling unit for all samples was five stems of alfalfa randomly selected one meter around the operator at each point, and the sample size was 20 units, 10 m apart from each other. The systematic sampling procedure followed the diagonals of the sampling area, taking 10 sample units in each diagonal. Because the edges tend to receive a greater number of eggs, a 15 m border was excluded from sampling. The five stems ofeach sample unit were bagged and then inspected in the laboratory for egg enumeration. The mean and variance were calculated for each sample. The relationship between variance and mean was characterized in a previous study (Serra et al. 2005) using Taylor's power law (Taylor 1961), based on an empirical relationship, and Iwao's patchiness regression (Iwao 1968), based on a deductive relationship (Kuno 1991).

### Sequential sampling plans

With the parameters obtained from regression analysis of Taylor's equation, Green's fixed precision sequential sampling plans were applied using the formula:


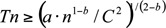


where *Tn* was the cumulative number of eggs, *n* the sample size, *C* the desired precision level in terms of standard error as a fraction of the mean, and *a* and *b* were the parameters of Taylor's power law (Green 1970). Similarly, with the parameters from Iwao's regression, enumerative sequential sampling plans were built where the stop-line was calculated by the formula proposed by Kuno (1968):


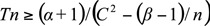


where *Tn* was the cumulative number of eggs, *n* the sample size, *C* the desired precision level, and α and β the parameters of Iwao's regression. The precision levels chosen for the sampling plans were *C* = 0.10 and *C* = 0.25, respectively. A precision level of 0.25 is generally accepted for sampling plans aiming at decision-making in pest management, while a precision level of 0.10 is recommended for studies of population dynamics (Southwood 1978).

The sequential sampling plans were validated using the Resampling for Validation of Sampling Plans (RVSP) developed by Naranjo and Hutchison (1997). Because RVSP requires the use of independent data sets for validation, 12 data sets were randomly selected, representing a range of low, medium, and high egg densities. These data sets were omitted when estimating the parameters of Taylor's power law and Iwao's patchiness regression, and were used only to validate the sampling plans. The RVSP was used for random resampling with replacement over the 12 data sets until the stop-line was achieved (Naranjo and Hutchison 1997). In addition to the fixed precision level of 0.10 and 0.25, a minimum sample size of five sample units was used for all the simulations. The resampling was repeated 500 times for each data set, which is adequate for most purposes (Naranjo and Hutchison 1997), yielding the average, minimum, and maximum precision levels and sample sizes.

## Results

Out of a total of 78 data sets, 11 had a single egg on a stem and were excluded from the analyses, since values of *S^2^* and *m* are restricted for singleton observations (*S^2^* = *m*), which leads to pseudo-randomness (Hamilton and Hepworth 2004). There were thus 67 data sets, 35 collected at FCA and 32 at Manfredi. Twelve data sets, with a mean ± SD of 1.71 ± 1.91 eggs per sample unit covering a range of mean densities of 0.35 to 7.2 eggs per sample unit, were separated for use as independent data sets for validation of sampling. The dispersion pattern was characterized with the remaining 55 data sets.

The mean ± SD density in the three years of observation was 1.21 ± 1.55 and 1.00 ± 1.74eggs per sample unit in FCA and EEA Manfredi, respectively. The minimum mean density was 0.10 eggs per sample unit in both locations, whereas the maximum mean density occurred on 15 February 2001 simultaneously in the two locations, with values of 6.35 and 8.35 eggs per sample unit for FCA and EEA Manfredi, respectively. These values are close to those observed by Aragón and Harcourt (1977a), who worked with a sample unit of three alfalfa stems and reported a range of average densities of 0.3 and 6.2 eggs per sample unit in from two seasons.

**Figure 1.**
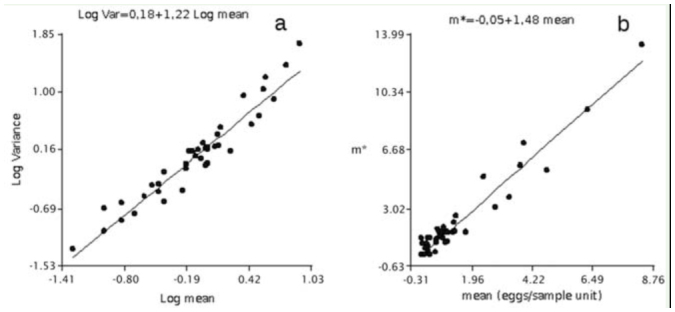
a) Relationship between log variance and log mean density of *Colias lesbia* eggs per sample unit on alfalfa fields (n = 55). b) Relationship between Lloyd's mean crowding and mean density of *C. lesbia* eggs per sample unit on alfalfa fields (n = 55). High quality figures are available online.

Taylor's power law parameters were: *a* = 1.51 and *b* = 1.22 (Figure 1a). The *b* value was significantly greater than 1 (*p* < 0.0001, *t* = 5.47, df = 53) (Figure 1a). Iwao's regression parameters were: α =-0.05 and β = 1.48 (Fig. 1b). The parameter α was not significantly different from 0 (*p* = 0.60, *t* = -0.52, df = 54), suggesting that the individual egg forms the basic component of aggregation (α +1). The parameter β was significantly greater than 1 (*p* < 0.0001, *t* = 10.11, df = 53). Both parameters *b* and β suggest the dispersion pattern of *C.* *lesbia* eggs is aggregated (Serra et al. 2005).

### Sequential sampling plans

**Figure 2.**
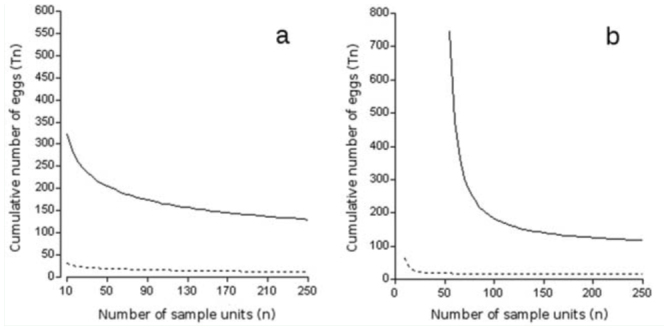
Sequential sampling plans for estimating the mean density of *Colias lesbia* eggs per sample unit on alfalfa crops with two precision levels: C = 0.10 (solid lines) and C = 0.25 (dashed lines), a) According to Green's model, b) According to Kuno's model. High quality figures are available online.

Green's sequential sampling plans (for *C* = 0.10 and *C* = 0.25) are presented in Figure 2a. To use the plan, a worker tracks the cumulative number of eggs (*Tn*) and the cumulative number of sample units (*n*) on the graph. Sampling ceases when the critical stop-line is intersected. The population density, determined as the mean number of eggs per sample unit (*Tn*/*n*), can then be calculated to the desirable precision level. The number of sample units required to estimate a hypothetical population of 1 egg per sample unit with a precision level of 0.1 and 0.25 will be 151 and 24, respectively. Resampling analysis with the RVSP software for a precision level of 0.10 resulted in an average sample size of 161 sample units, with a range of 33 to 349 sample units (Table 1). For the 12 data sets covering different densities, the actual average precision level was 0.10, which is equal to the desired precision for population research. Similarly, for a prefixed precision level of 0.25, the resampling analysis resulted in an average sample size of 27, with a range of 6 to 59 sample units. The average precision level of this analysis was 0.24, which is close to the preset precision of 0.25 (Table 1).

**Table 1.**
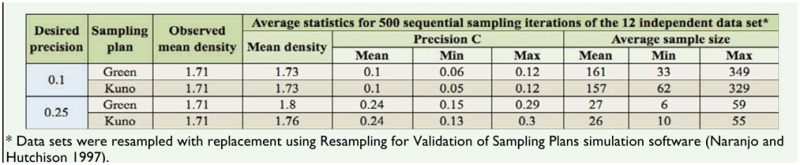
Resampling results for validation of Green's and Kuno's enumerative sequential sampling plans, using desired fixed-precision levels of 0.10 and 0.25 to estimate the density of *Colias lesbia* eggs on alfalfa crop.

**Figure 3.**
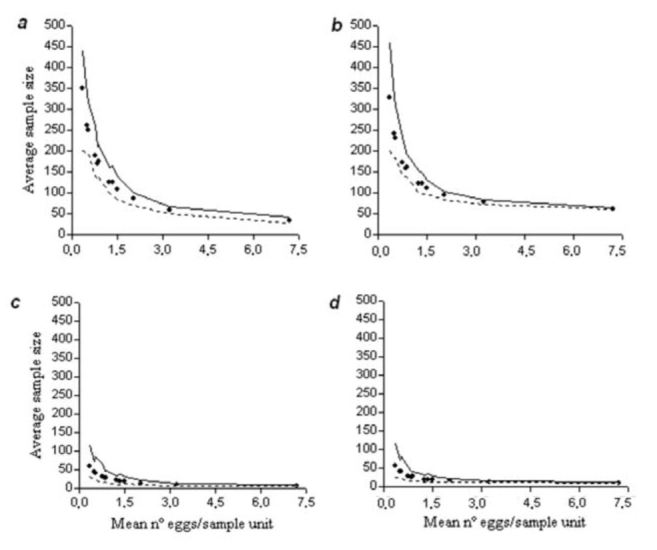
Summary of resampling validation analysis using 12 independent data sets of *Colias lesbia* eggs on alfalfa showing the average sample size for Green's sequential sampling plan (graphs *a* and *c*) and for Kuno's sequential sampling plan (graphs *b* and *d*). Precision levels for the sampling plans were: C = 0.10 (graphs *a* and *b*) and C = 0.25 (graphs *c* and *d*)*.* Black circles denote mean values; solid lines and dashed lines denote the maximum and minimum values, respectively. High quality figures are available online.

**Figure 4.**
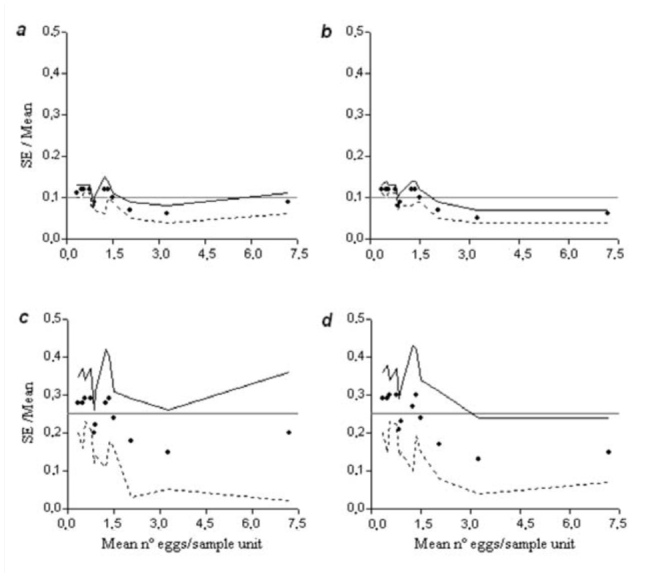
Summary of resampling validation analysis using 12 independent data sets of *Colias lesbia* eggs on alfalfa showing the average precision levels (C = SE/Mean) for Green's sequential sampling plan (graphs *a* and *c*) and for Kuno's sequential sampling plan (graphs *b* and *d*). Precision levels for the sampling plans were: C = 0.10 (graphs *a* and *b*) and C = 0.25 (graphs *c* and *d*). Horizontal lines in *a* and *b* represent the desired precision level of 0.10, and horizontal lines in *c* and *d* represent the desired precision level of 0.25. Black circles denote mean values; solid lines and dashed lines denote the maximum and minimum values, respectively. High quality figures are available online.

Sample size requirements for the desired precision level of 0.10 and 0.25 declined rapidly with increasing mean density of eggs. Furthermore, as higher precision is used to characterize the mean, a greater sample size is necessary (Figure 3 a, c). The validation analysis using the RVSP software showed acceptable precision of sampling plans developed for both ecology studies and pest management plans (Figure 4 a, c; Table 1).

Kuno's sequential sampling plans (for *C* = 0.10 and *C* = 0.25) are presented in Figure 2b. The stop-lines obtained with the parameters of Iwao's regression, similar to those described in the sampling plan presented above, relate the cumulative number of eggs to a corresponding number of sample units. In this case, 143 and 23 sample units would be required to estimate a hypothetical population of 1 egg per sample unit with a precision level of 0.1 and 0.25, respectively.

Resampling analysis for a prefixed precision level of 0.10 resulted in an average sample size of 157 sample units, with a range of 62 to 329 (Table 1). The average precision level of 0.10 matched the desired precision. Similarly, for a prefixed precision level of 0.25, the resampling analysis gave an average sample size of 26, with a range of 10 to 55 sample units. The average precision level of this analysis was 0.24, which is close to the prefixed precision of 0.25 (Table 1).

As with Green's sampling plan, the average sample size declined rapidly with increasing mean density of eggs, i.e., when a higher precision was used to estimate the mean density, a greater sample size is necessary (Figure 3 b, d). The validation analysis using the RVSP software showed a good precision of the sampling plans developed using this methodology for both ecology studies and pest management plans (Fig. 4 b, d; Table 1).

## Discussion

The sequential sampling plans obtained with both methods yielded very similar average required sample sizes (Table 1), making it difficult to choose one or the other for field application. A first consideration suggests a slight advantage in the use of Kuno's sequential sampling plan, because it requires a lower average sample size to reach the stop-line at the two precision levels evaluated. However, the comparison of methods in terms of sensitivity to changes in egg density showed that, at intermediate egg densities (i.e., higher than 1.5 eggs per sample unit), Green's plan requires fewer sample units than Kuno's to reach the decision line. This becomes even more noticeable at high densities. For example, to estimate a population mean of 5 eggs per sample unit, with a precision level of 0.10, 43 and 67 sample units were required with Green's and Kuno's sampling plans, respectively, and if the density was 8.35 eggs per sample (the maximum mean density observed in this work), it would take 29 and 59 sample units to reach the stop-line for Green's and Kuno's sequential sampling plans, respectively. Therefore, if the end user needs a plan with an acceptable trade-off between time invested in sampling and the precision of the resulting estimation in the whole range of possible densities found in the field, we would recommend using the stop-line of Green's sequential sampling plan.

Similar fixed precision sequential sampling plans have been developed and validated using the resampling methodology proposed by Naranjo and Hutchison (1997) for different insect species, including eggs, nymphs, and adults of whiteflies in sweet potato (Naranjo and Hutchison 1997), larvae of *Ostrinia nubilalis* (Hübner) and *Heliothis zea* (Boddie) in corn (O'Rourke and Hutchison 2003), adults and larvae of the predatory ladybird beetle, *Harmonia axyridis* (Pallas), in sweet corn (Koch et al. 2006), and *Helicoverpa* sp. eggs in tomato (Dawson et al. 2006). This approach shows that, when adequate independent data sets are used for validation, the resulting sequential sampling plan can be reliably used to ensure that the desired fixed-precision levels are achieved (O'Rourke and Hutchison 2003).

The sequential sampling plans presented in this paper are valid for use in alfalfa fields located in the province of Cordoba, Argentina, and will provide researchers with a sampling tool suitable for addressing issues in ecology and population dynamics related to *C. lesbia* eggs. From the standpoint of integrated pest management, these sampling protocols could provide a quick method to make control decisions based on the abundance of eggs, e.g., decision-making on inundative release of parasitoids of *C. lesbia* eggs. However, its implementation requires further research on various aspects of parasitoid-pest relationships. Studies on population dynamics are also needed to transfer the action threshold of 0.6 larvae per stem to the number of eggs, i.e., how many eggs per sample unit are required to later produce an infestation of 0.6 larvae per stem?

## Abbreviations

Bt: *Bacillus thuringiensis*
RSPV: Resampling for Validation of Sampling Plans (software)
